# BGP‐15 Treatment Improves Follicle Growth and Alters Collagen Deposition in Mouse Transplanted Ovaries

**DOI:** 10.1002/mrd.70128

**Published:** 2026-07-11

**Authors:** Luiza Aparecida Ansaloni Chagas Pereira, Larissa Aline Freitas, Camila Stefane Ferreira, Karine Stephany Serpa Amaral Dias, Paulo Henrique Almeida Campos‐Junior

**Affiliations:** ^1^ Laboratory for Reproductive Biology Research, Department of Natural Sciences Federal University of São João del Rei. São João Del‐Rei Minas Gerais Brazil

**Keywords:** BGP‐15, collagen deposition, follicular outcome, ovarian transplantation

## Abstract

Ovarian transplantation causes a loss of follicular reserve, requiring new strategies to optimize post‐transplant follicular growth. This study investigated *N‐(2‐hydroxy‐3‐piperidin‐1‐ylpropoxy)pyridine‐3‐carboximidamide; dihydrochloride* (BGP‐15), a mitochondrial modulator, as a follicular growth stimulator and a collagen‐depositing inhibitor, thereby improving graft quality. In vitro, neonatal mouse ovaries were treated with different concentrations of BGP‐15. In vivo, adult mice undergoing ovarian autotransplantation received 10 mg/kg of BGP‐15. Follicular quantification, gene expression by qRT‐PCR (*Sirt1*, *Sirt3*, *Nampt*, *Foxo3a*, *KitL*, *Pten*, *Amh*), collagen deposition by Picrosirius assay, neoangiogenesis, macrophage infiltration, biometric parameters, and the estrous cycle were evaluated. In vitro, 0.001 µM BGP‐15 induced follicular growth. reducing the percentage of primordial follicles (*p* < 0.0001) and upregulating *Sirt1* (*p* < 0.0001), *Sirt3* (*p* = 0.026), *Nampt* (*p* = 0.002), *Pten* (*p* = 0.012), and downregulating *Amh* (*p* = 0.031). In vivo, BGP‐15 treatment in transplanted mice resulted in an earlier resumption of the estrous cycle (*p* = 0.0458). After transplantation, BGP‐15 improved follicular outcome on day 22, increasing healthy follicles (transitional *p* = 0.0206; primary *p* = 0.0125; secondary *p* = 0.0428) and reducing atretic follicles (*p* = 0.0381). Treated animals exhibited decreased macrophage numbers (*p* < 0.0001) and collagen deposition (ovary: *p* = 0.0465; capsule: *p* = 0.0344), and increased blood vessel density (*p* < 0.0001). BGP‐15 improves follicular growth and survival in ovarian grafts, coinciding with mitochondrial gene dysregulation and reduced collagen deposition. These findings suggest BGP‐15 as a promising candidate for improving follicular outcomes after ovarian transplantation.

## Graphical abstract

Ex vivo treatment with BGP‐15 promotes follicular activation and gene regulation (*Sirt1*, *Sirt3*, NAMPT, *Pten*, *Amh*). After transplantation, BGP‐15 reduces atresia and collagen deposition, stimulating follicular growth, survival, and neoangiogenesis at timepoints of 6 and 22 days post‐transplantation.

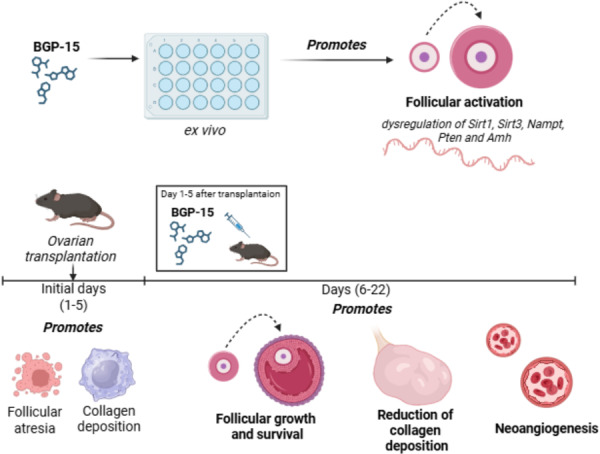

## 1 Introduction

Among the proposed strategies for fertility preservation, ovarian transplantation has emerged as an alternative with more than 200 live births reported (Lee et al. 2017; Fabbri et al. 2024). However, this results in a depletion of the follicular reserve due to the massive activation of follicles (transition from primordial to primary follicles) and subsequent atresia (Milenkovic et al. 2012; Gavish et al. 2018; Bertoldo et al. 2018; Nascimento et al. 2023). For patients with few follicles, new procedures must be standardized to stimulate the remaining follicles. In vitro follicular activation techniques have been developed to recover follicles from cryopreserved ovarian tissue, utilizing pharmacological agents and mechanical or chemical manipulation (Bertoldo et al. 2018; Fàbregues et al. 2021; Hu et al. 2023). In this context, mitochondria play a central role in ovarian function, particularly during follicular activation and growth, providing energy for cell proliferation and maintaining redox homeostasis (Duran et al. 2011; Umehara et al. 2022). Furthermore, genes related to mitochondrial biogenesis and function, such as Sirtuins 1, 3, and Nicotinamide Phosphoribosyltransferase, regulate cell survival and resistance to oxidative stress, proving crucial for follicular quality and, recently, being associated with follicular activation (Zhang et al. 2019; Zhao et al. 2021).

Neoangiogenesis is a crucial process for the establishment of ovarian grafts, as it enables revascularization of the transplanted tissue, thereby reducing ischemia and acute follicular loss during the early post‐transplant phase (Nascimento et al. 2023). A delay in the formation of new vessels compromises the follicle survival and growth, making strategies that accelerate angiogenesis (e.g., angiogenic factors, endothelial cells, or biomaterials) crucial (Zaninović et al. 2024). On the other hand, excessive collagen deposition and pathological remodeling of the extracellular matrix (fibrosis) directly alter the biomechanics and microenvironment of the ovary, impairing follicular growth, post‐ovulation healing, and regulating oocyte release (Gu et al. 2024). Ovarian fibrosis is associated with symptoms of ovarian dysfunction, including reduced ovulatory capacity and a decline in oocyte quality (Gu et al. 2024). Thus, the combination of rapid restoration of vascular support and control of collagen deposition/fibrosis constitutes a central therapeutic agent for improving follicular survival and hormonal and reproductive function in cases of ovarian problems.

BGP‐15 [(O‐[3‐piperidino‐2‐hydroxy‐1‐propyl]‐nicotinic amidoxime)] has been demonstrated to be an insulin sensitizer (Literáti‐Nagy et al. 2012) and a stimulator of mitochondrial function (Gonzalez et al. 2025). It was previously shown that mitochondrial dysfunction leads to ovarian fibrosis, impairing ovulation in aged and obese females (Umehara et al. 2022), aspects not yet investigated in transplanted ovaries; in aged females, BGP‐15 reduced ovarian collagen deposition and restored ovulatory capacity (Umehara et al. 2022), opening new perspectives for the use of this drug in other contexts of ovarian biology. Accordingly, it remains unclear what the dynamics of collagen deposition are and whether BGP‐15 treatment can modulate follicular growth and fibrosis in the context of ovarian transplantation.

Because BGP‐15 is a new drug not yet approved by the Food and Drug Administration (FDA), and pharmacokinetic studies are not yet available, laboratory‐based studies must be conducted. Thus, the present study aimed to investigate, in a pre‐clinical assay, the efficacy of BGP‐15 treatment as a stimulator of follicle growth (ex vivo and after transplantation) and as a modulator of collagen deposition in transplanted ovaries, thereby improving folliculogenesis.

### 1.1 Materials and Methods

#### 1.1.1 Experimental Animals

Female C57Bl6J 3‐day‐old and 6‐week‐old mice, from the Laboratory Animal Breeding Center of the Federal University of São João del‐Rei (NUCAL ‐ UFSJ), were used. Neonates were used for ex vivo and gene expression assays, while 6‐week‐old mice were used for transplantation procedures. The animals were maintained in a 12/12‐h photoperiod with ad libitum access to water and food, and the Ethics Committee approved all experiments for the Use of Laboratory Animals (CEUA) at UFSJ (protocol number 6986150322).

#### 1.1.2 Ex Vivo Experiment

**Figure 1:**
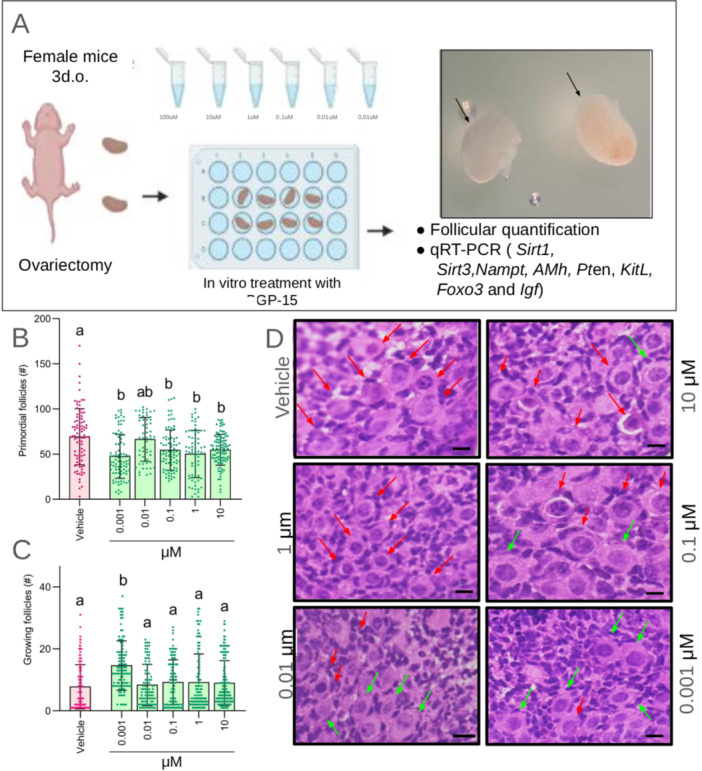
(A) Experimental design. For *in vitro* treatment, 3‐day‐old female animals were used. The ovaries were collected and treated in vitro for follicular quantification and gene expression analyses. Follicular activation was altered by in vitro treatment with BGP‐15. The drug was tested in a serial dilution for the in vitro assay at concentrations of 10, 1, 0.1, 0.01, and 0.001 μM. The experimental group consisted of 6 ovaries each. The samples treated with the drug exhibited fewer primordial follicles per section, except at 0.01 μM (B). On the other hand, a greater number of activated follicles was observed at the lower concentration (0.001 μM) than in the other groups (C). Different letters represent statistical differences between the groups. Histological sections (D) corroborate the statistical analysis. A greater percentage of growing follicles (transitional, primary, and secondary ‐ green arrows) at the 0.001 µM concentration, in contrast to a lower percentage of primordial follicles (red arrows). Bars = 200 μm.

Postnatal Day 3 (PD3) mouse ovaries were used because this stage marks a critical period of germ cell cyst breakdown, primordial follicle formation, and early follicular activation (Reddy et al. 2008; Adhikari et al. 2009; Hsueh et al. 2015). This methodology was also previously standardized by our group (Oliveira et al. 2026). Dissection medium ‐ penicillin‐streptomycin (15140122, Gibco, Carlsbad, CA, United States), Fetal Bovine Serum (FBS ‐ Sigma‐Aldrich, F2442, United States), and Phosphate‐buffered Saline (PBS ‐ Nova Biotecnologia, 133026205, São Paulo, Brazil) and growth medium ‐ DMEM/Ham's F‐12 culture medium – enriched with l‐Glutamine and HEPES [3.5 g/L] (NovaBio, BR30004‐05, São Paulo, Brazil); bovine serum albumin (BSA) (Sigma‐Aldrich, A9418, United States), and insulin‐transferrin‐selenium (ITS) 100× (Sigma‐Aldrich, I3146, United States) were prepared. 24‐well plates were prepared with 400 µL of growth medium, and a Transwell (Millipore Sigma, PICM01250, Darmstadt, Germany) was added for the ovarian culture. The surrounding wells were filled with 500 µL of filtered PBS. The plates were incubated at 37°C for 1 h before the ovaries were isolated (Figure 1A).

To isolate the ovaries, neonatal female mice were euthanized, and both ovaries were removed and transferred to a 35 mm dish containing the dissection medium at 37°C. Under a stereoscopic microscope (Zeiss Stemi 508, Jena, Germany), the adjacent structures were removed using insulin syringes. The ovaries were placed in the Transwell containing the equilibrium medium, and subsequently, an additional 100 µL of spare growth medium was added to the tissue. Plates were incubated for 3 days. Subsequently, the ovaries were encapsulated in alginate, stained with Alcian Blue (5% ‐ Sigma‐Aldrich, A5268, São Paulo, Brazil), fixed in 4% paraformaldehyde (Neon, 3899, São Paulo, Brazil) for 24 h, and then maintained in 70% alcohol for histological preparation. The drug was diluted in Phosphate‐Buffered Saline solution to its molar mass (BGP‐15: 315.3 g/mol; Sigma‐Aldrich, B4813, Darmstadt, Germany), and serial dilutions were prepared: 10, 1, 0.1, 0.01, and 0.001 µM. Vehicle ovaries were incubated only with the growth medium, and six ovaries were used for each group (Figure 1A ). The ovaries were directed for standard histological processing and stained with hematoxylin‐eosin (H&E) for follicular quantification and classification (Pereira et al. 2020). Histological sections were prepared at 5 μm from mouse ovaries, as described by Zheng and collaborators (2021). All ovarian sections were counted, and only follicles containing an oocyte with a clear nucleus were considered to avoid double‐counting. For the *ex vivo* assay, follicles were shown as the number of follicles per section. For this assay, “growing follicles” represent the number of transitional + primary follicles.

### 1.2 Gene Expression by qRT‐PCR

About 0.001 μM was considered the most effective concentration for follicular activation, as it showed a reduced number of primordial follicles, contrasting with an increased number of activated follicles. Therefore, 0.001 μM was chosen for quantitative real‐time PCR (qRT‐PCR) to investigate whether in vitro treatment with BGP‐15 alters the expression of target genes. RNA extraction was performed on both the Vehicle and BGP‐15 ovaries using the InviTrap Spin Universal RNA Mini Kit (1060100200 ‐ Invitek Diagnosis, Berlin, Germany). Due to the small size of the analyzed organs, four pools of 9 ovaries from each group (*n* = 36 ovaries) were prepared, and 1 μg of RNA was converted to cDNA using the High Capacity cDNA Reverse Transcription Kit (containing 5× iScript Reaction Mix and iScript Reverse Transcriptase 1708890 ‐ Bio‐Rad, Hercules, CA, United States). For all reactions, the following parameters were used: 50°C and 95°C for 2 min each, and 45 cycles of 95°C for 15 s, 60°C for 30 s, and 72°C for 20 s. For this, the QIAquant‐2 plex Machine (Qiagen, Germany) and iTAq™ Universal SYBR Green Supermix (1725120, Bio‐Rad, Hercules, CA, United States) were used. Target genes were: *Sirt1* (Sirtuin 1), *Sirt3* (Sirtuin 3), *Nampt* (Nicotinamide phosphoribosyltransferase), *Foxo3a* (Forkhead Box O3a), *KitL* (KIT proto‐oncogene, receptor tyrosine kinase), *Pten* (Phosphatase and Tension Homolog), *Amh* (anti‐Müllerian hormone), *Igfr* (Insulin‐like growth factor 1 receptor), and the β‐actin gene (*Actb*) as an endogenous reference, being this expressed constitutively even in mouse ovaries. Their sequences and other details are provided in Table 1. Dissociation was performed at the end of amplification to verify the primer melting temperature, which was used to confirm its specificity. All procedures followed the manufacturer's instructions. REST© 2009 software was used for analysis (Pfaffl et al. 2002). Ovaries from control groups were used to calibrate the relative transcription levels, which were reported as n‐fold differences relative to the calibrator, and the error bars represent the standard error of the mean (SEM). All these procedures have already been performed and previously described (Pereira et al. 2020; Nascimento et al. 2022; Nascimento et al. 2023).

**Table 1:** Primer sequences used in qPCR analysis. Genes related to mitochondrial activation (*Sirt1, Sirt3*, and *Nampt*), ovarian reserve (*Amh*), follicle activation and folliculogenesis (*Foxo3a*, *KitL*, and *Pten*), and the endogenous control gene beta actin (*Actb*). The table represents their respective primers ‐ Forward and Reverse(5′‐3′), annealing temperature (°C), and the accession code from NCBI.

Genes	Primers sequences (5′ – 3′)	Annealing Temperature (°C)	Access Code
*Sirt1*	F: AAGTTTTGTGTTTGGGTGCG	58,5	NM_019812.3
	R: GATCTCTGCCAAGTTCCAGG		
*Sirt3*	F: GGTTCCCTTGGGTGTCATTT	60	NM_001127351.1
	R: GAGTGACATTGGGCCTGTAG		
*Amh*	F: TGTGTGTAAAGAAGGGTGTTTGTR: AAAGCAAGCAAACACACACA	60	NM_007445.3
*Nampt*	F: GTAACCTTCCTGCCTTCCCT	60	NM_021524.2
	R: ACCGCCCACAAGTCAGATAA		
*Foxo3a*	F: GGGTCCCACAGCAACGATG	60	NM_001376967.1
	R: CACCAGGGAATGCACGTCC		
*Kitl*	F: GAATCTCCGAAGAGGCCAGAA	60	NM_013598.4
	R: GCTGCAACAGGGGGTAACAT		
*Pten*	F: TGGAGCCTGCATTTGGAAGT	60	NM_008960.2
	R: TCAAGTGTGGCGAGGTCAAA		
*Actb*	F: TGGCACCACACCTTCTACAA	60,7	NM_007393.5
	R: ATGGGAGAACGGCAGAAGAA		

### 1.3 Ovarian Transplantation and the Assessment of the Post‐Transplant Initial Days

This experiment was performed to evaluate the collagen deposition and follicle survival during the first days after subcutaneous transplantation. Animals were subjected to inhalant anesthesia (Isoflurane ‐ Abcam ‐ ab145581, Rio de Janeiro, Brazil) and ovariectomy. Ovaries were subcutaneously autotransplanted as previously detailed (Nascimento et al. 2022). To evaluate the follicle survival and collagen deposition in the initial days after subcutaneous transplantation, euthanasia was performed 12 h, 1 d, 2 d, 3 d, 4 d, and 5 d (*n* = 4 for each group) after this procedure. For follicular quantification, ovaries were collected, and follicle quantification was performed as previously described (Pereira et al. 2020). In this case, follicle quantification is expressed as the number of follicles per section. Follicle quantification was performed using serial section analysis to ensure an accurate and unbiased assessment of follicle populations. Ovarian tissues were fixed, embedded, and sectioned (5 µm). Serial sections were obtained throughout the entire ovary, and follicle counting was conducted systematically across all sections to avoid overestimation and double‐counting. Only follicles with a clearly visible oocyte nucleus were included in the analysis. Follicles were classified according to established morphological criteria into primordial, transitional, primary, and secondary stages. This approach represents a standard procedure in our laboratory and was specifically adopted to minimize bias and improve the reliability of follicle quantification across different developmental stages.

To evaluate collagen deposition in transplanted ovaries, middle sections of the transplanted ovaries (from the previously cited time points ‐ 5 per ovary) were stained with Picro‐Sirius Red (PSR) (EP1120011A ‐ EasyPath Diagnosis, São Paulo, Brazil) according to the manufacturer's instructions. The PSR‐stained area was evaluated using ImageJ software, as previously described (Mara et al. 2020; Umehara et al. 2022). This data is expressed as PSR area in pixels/μm^2^.

### 1.4 *In Vivo* Treatment With BGP‐15

BGP‐15 is a drug not yet approved by the FDA, and there are no studies in the literature on its half‐life and pharmacodynamics. Based on our in vitro studies, 0.001 μM was the most effective dose to stimulate follicle growth, corresponding to 3.5 × 10^−7^ mg/kg, which would be a much lower concentration than those previously tested (Crul et al. 2013; Literáti‐Nagy et al. 2012; Literáti‐Nagy et al. 2014; Umehara et al.^2022^. Therefore, 10 mg/kg was used in this study, which has been reported to be effective in increasing insulin sensitivity in rabbits and rats (Literáti‐Nagy et al. 2014), and is 10 times lower than the dose reported to reduce collagen deposition during murine ovarian aging (Umehara et al. 2022).

BGP‐15 (10 mg/kg) was administered (i.p.) (Literáti‐Nagy et al. 2012; Literáti‐Nagy et al. 2014) daily for 5 days, always at 9:00 AM. As the vehicle, PBS was administered. Treatment was started on the same day as ovarian transplantation, which was performed as previously described (Nascimento et al. 2023). Animals were euthanized at two different time points, as the initial days after transplantation are already known to be the most important for follicular survival in mice; 6 days is a critical time point to investigate the effects of BGP‐15 on maintaining the ovarian pool and quality after transplantation. On the other hand, in mice, the complete duration of folliculogenesis (from primordial to antral follicles) is approximately 21 days. In this sense, 22 days are essential for evaluating whether the treatment administered immediately after transplant altered follicle growth to the antral stage. Ovaries were collected, fixed, and submitted for histological processing (Figure 5A). Follicles were classified as described for in vitro experiments; however, at the 22‐day time point, antral follicles were also considered. Follicular quantification was performed as previously described (Pereira et al. 2020) and is expressed as the number of follicles per section.

Body weight was measured throughout the experimental period. The estrous cycle was also measured to assess the ability of the transplanted ovaries to restore endocrine function. This parameter was evaluated every morning (9:00 AM) through vaginal cytology as previously described (Byers et al. 2012). The following parameters were assessed: (1) cycle length ‐ quantification of the number of days required for the animal to leave one proestrus and enter another proestrus, (2) cycle restart (days) ‐ number of days required for the transplanted animals complete one cycle after the transplantation, (3) days in each stage (%) ‐ percentage of days spent in each stage of the estrous cycle considering the entire experiment (22 day).

Immunohistochemistry for markers of blood vessels (CD31 ‐ 77699 ‐ Cell Signaling Technology, Danvers, USA) and macrophage (F4/80 ‐ 30325 ‐ Cell Signaling Technology, Danvers, USA) was performed. After deparaffinization, the tissue slides were boiled for 5 min in a citrate buffer (Neon, São Paulo, Brazil/0.01 M, pH 6) for antigen recovery. All immunostaining procedures were performed using the Vectastain® Elite® ABC Universal Plus Kit, Peroxidase (PK8200 ‐ Vector Laboratories, Newark, CA, United States), and sections were counterstained with hematoxylin. The slides were then analyzed under an optical microscope, and images were obtained at 100x and 400x magnification. The analyses were performed using ImageJ. For CD31, blood vessels with up to three CD31‐positive endothelial cells were identified and quantified in 25 photomicrographs per group, at the middle and periphery of the organ, at 400x magnification, yielding the number of blood vessels per 250,000 μm^2^. For macrophage quantification, the number of F4/80+ cells was determined for each group using 40 photomicrographs at a 400× magnification, with images taken from the middle and periphery of the organ. For statistical analysis, the number of positive F4/80 cells per 250,000 μm^2^ (Li et al. 2022) was used. For the PSR assay, 10 whole‐ovary samples from each group on day 22 were stained and analyzed as described; data are expressed as PSR‐positive area (pixels/μm^2^).

### 1.5 Statistical Analyses

Quantitative data were tested for normality using the D'Agostino & Pearson and Shapiro–Wilk tests. Parametric data were subjected to analysis of variance followed by the Bartlett test (dose‐response curve, initial days of follicular quantification) or an unpaired *t*‐test (body weight, days in each stage of the estrous cycle, follicular quantification, PSR, blood vessels, and macrophage quantification). Regarding nonparametric data, the Mann–Whitney test (initial days PSR) and the Whitney test (cycle length and cycle restart) were used, both with a significance level of *p* < 0.05. All analyses were performed using GraphPad Prism 8.0.

## 2 Results

### 2.1 Ex Vivo Treatment With 0.001 μm of BGP‐15 Induced Follicle Activation and Dysregulated Some Key Genes

Follicular quantification demonstrated that treatment with BGP‐15 modulated the primordial follicle pool in neonatal ovaries cultured in vitro. The Vehicle group showed a significantly higher number of primordial follicles compared with the 0.001, 1, and 10 µM groups (*p* < 0.05), while the 0.01 and 0.1 µM concentrations showed intermediate values, not differing statistically from either group (0.001 µM, *p* < 0.0001; 0.1 µM, *p* = 0.0009; 1 µM, *p* < 0.0001; 10 µM, *p* = 0.0006; ‐ Vehicle 69.27 ± 3.2; 0.001 µM, 47.66 ± 2.6; 0.1 µM, 54.5 ± 2.4; 1 µM, 50.2 ± 3.1; 10 µM, 54.2 ± 1.7). Regarding growing follicles, only the 0.001 µM concentration significantly increased the number of developing follicles compared with the Vehicle group (*p* < 0.05 ‐ Figure 1B,D), whereas the remaining concentrations showed no significant differences (transitional + primary ‐ *p* < 0.0001; Vehicle 7.7 ± 0.5; 0.001 µM, 14.6 ± 0.7; 0.01 µM, 8.3 ± 0.7; 0.1 µM, 9.2 ± 0.6; 1 µM, 9.1 ± 0.8; 10 µM, 8.9 ± 0.5; Figure 1C,D).

**Figure 2:**
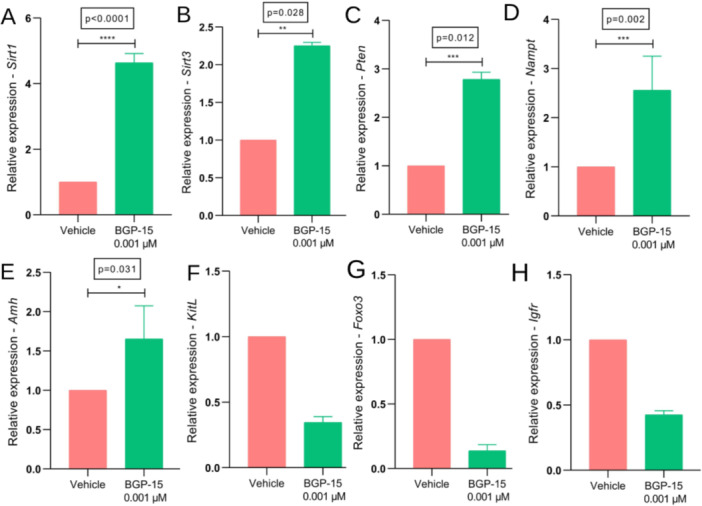
In vitro treatment with 0.001 μM of BGP‐15 changed the relative expression of genes associated with follicular and mitochondrial activation. The ovaries of the treated group showed an increase in the relative expression of *Sirt1* (A, *p* < 0.0001), *Sirt3* (B, *p* < 0.028) and *Nampt* (C, *p* = 0.002), besides *Pten* (D, *p* = 0.012) and *Amh* (F, *p* = 0.031). For the *Kitl* (G), *Foxo3* (H), and *Igf1* (I) genes, no significant differences were observed (*p* > 0.05). Asterisks indicate statistical differences and erroR bars represent the standard error of the mean (SEM).

In the BGP–15–treated ovaries, there was an upregulation of mitochondrial activation genes, including *Sirt1* (*p* < 0.0001, Figure 2A), *Sirt3* (*p* = 0.028, Figure 2B), and *Nampt* (*p* = 0.002, Figure 2C), indicating a potential enhancement of mitochondrial biogenesis and metabolic activity. BGP‐15 treatment upregulated *Pten* – a well‐known inhibitor of follicle activation (*p* = 0.012, Figure 2D) and *Amh* – a factor related to the initial follicle growth (*p* = 0.031, Figure 2E). Conversely, no significant differences (*p* > 0.05) were detected in the expression of *Kitl* (Figure 2F), *Foxo3* (Figure 2G), and *Igfr* (Figure 2H).

### 2.2 The First Days After Transplantation are Crucial to Follicle Survival and Collagen Deposition

**Figure 3:**
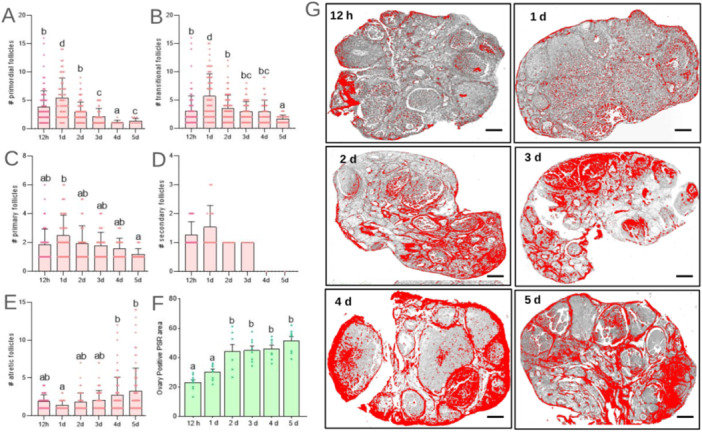
Initial post‐transplantation days are the most crucial for follicular survival. Transplanted ovaries show a reduction of the number of healthy follicles in different classes of development (primordial‐A, transitional‐B, primary‐C, and secondary‐D), contrasting with an increasing number of atretic follicles (E). Collagen deposition becomes more noticeable on the second day after transplantation and is very prominent by day 5 (F). The histology (G) demonstrates the progressive collagen deposition in the area marked by PSR. Bars = 100 μm.

During the first 5 days post‐transplant, the number of primordial follicles (Figure 3A) was reduced on day 3 compared to 12 h (*p *< 0.0001) and to day 1 (*p* < 0.0001). On day 4, the number of primordial follicles was reduced compared to 12 h (*p* = 0.0016), day 1 (*p* < 0.0001), and day 2 (*p* < 0.0001). On the 5th day, primordial follicles were also reduced compared to 12 h and day 1 (*p* = 0.0047 and *p* < 0.0001 ‐ 12 h, 3.9 ± 2.3; 1 day, 5.4 ± 3.5; 2 day, 2.9 ± 1.7; 3 day, 2.1 ± 1.4; 4 day, 1.1 ± 0.3; 5 day, 1.3 ± 0.5). The number of transitional follicles (Figure 3B) was reduced on day 2 compared to day 1 (*p* = 0.041), and on day 3, the number of transitional follicles was reduced compared to day 1 (*p* = 0.0037). The number of transitional follicles was decreased on day 4 compared to day 1 (*p* = 0.0006), and on day 5, the number of transitional follicles was reduced on 12 h (*p* = 0.0475), 1 day (*p* < 0.0001), 2 day (*p* = 0.0007) and 3 day (*p* = 0.0245 ‐ 12 h, 3.0 ± 2.7; 1 day, 5.6 ± 3.9; 2 day, 3.5 ± 1.9; 3 day, 2.9 ± 1.8; 4 day, 2.9 ± 2.1; 5 d, 1.6 ± 0.7). The number of primary follicles (Figure 3C) was reduced on day 5 compared to day 1 (*p* < 0.0001 ‐ 1 d 2.8 ± 1.4, 5 d 1.2 ± 0.3). The number of secondary follicles was not altered (Figure 3D); however, the presence of this follicular class was only observed up to day 3 post‐transplant. As expected, no secondary and antral follicles were observed on days 4 and 5 post‐transplant. In general, the number of follicles was reduced by 91.02% on the 5th day compared to day 1. Conversely, the number of atretic follicles (Figure 3E) was increased on day 5 compared to days 1 (*p* = 0.0055) and 2 (*p* = 0.0014; 1 day, 1.4 ± 0.6; 2 day 1.8 ± 1.2; 5 day, 3.2 ± 3.0).

On the other hand, a significant increase in collagen deposition was observed from the second day post‐transplant (Figure 3F). The positive PSR area increased on days 5 (*p* = 0.0080), 4 (*p* = 0.0039), 3 (*p* < 0.0001) and 2 (*p* = 0.0148) compared to day 1 and to 12 h (*p* < 0.0001; 12 h 23.2 ± 5.0; 1 day, 30.5 ± 5.1; 2 day, 44.1 ± 13.3; 3 day, 45.0 ± 8.0; 4 day, 46 ± 6.9; and 5 day, 51.5 ± 8.2). More specifically, the PSR marking increased by 46.02% on day 5 compared with the 12 h time point. Corroborating the statistical analysis, the photomicrograph shows a progressive increase in PSR marking over the evaluated time period (Figure 3G).

### 2.3 Biometric Parameters and the Estrous Cycle of Ovarian Transplanted Animals were Not Affected by the Treatment With BGP‐15

**Figure 4:**
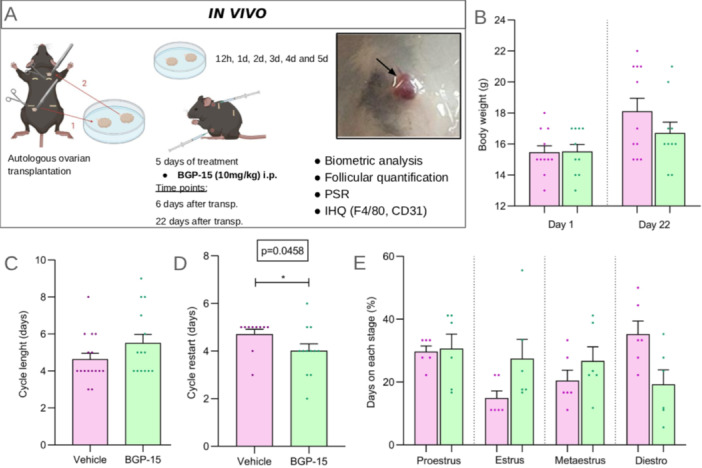
For *in vivo* treatment (A), ovaries from adult female animals were recovered, transplanted, and collected after the timepoints of 12 h, 1 day, 2 day, 3 day, 4 day, or 5 day. Treatment was performed for 5 days with BGP‐15 at a concentration of 10 mg/kg. After treatment and at the respective timepoints of euthanasia (6 or 22 day), the ovaries were collected and processed for histological preparation and analysis of follicular quantification, morphometry, and immunohistochemistry. PSR quantification and immunostaining for F4/80 and CD31 were performed. BGP‐15 treatment did not affect body weight on the 22nd day (B). Cycle length (C) was not significantly altered. BGP‐15‐treated animals showed an earlier cycle restart (D) than Vehicle‐treated animals (*p* = 0.0458). No statistical differences were observed in the frequency of days across the estrous cycle (E).

Figure 4A illustrates the experimental design for the in vivo experiments. Post‐transplantation, BGP‐15 did not alter body weight in treated animals (*p* > 0.05; Figure 4B). Treated animals resumed their estrous cycle earlier than the vehicle (*p* = 0.0458; Vehicle: 4.7 ± 0.2134; BGP‐15: 4.0 ± 0.3015; Figure 4C). Furthermore, BGP‐15‐treated animals showed similar outcomes for estrous cycle length (*p* > 0.05; Figure 4D) and the percentage of days in each stage (*p* > 0.05; Figure 4E).

### 2.4 In Vivo Treatment With BGP‐15 After Ovarian Transplantation Induced Follicle Growth and Collagen Deposition

**Figure 5:**
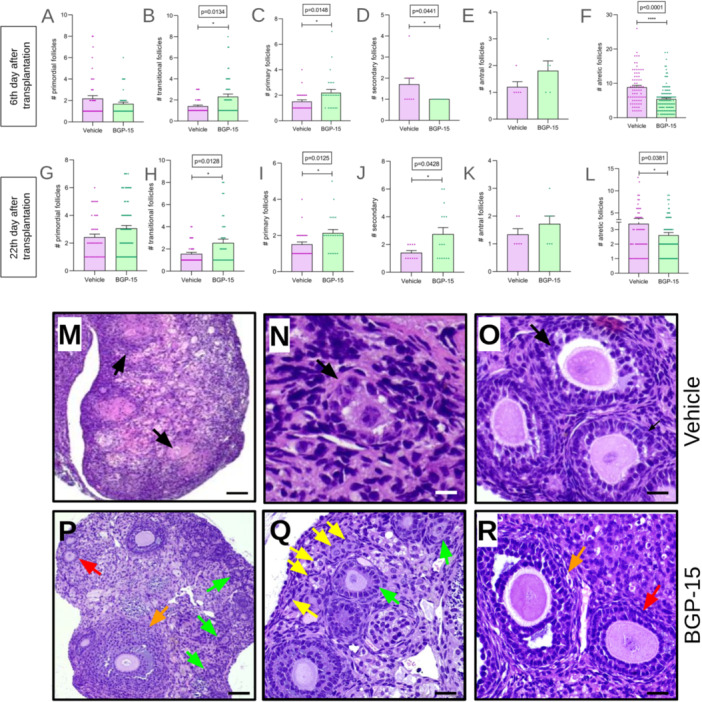
BGP‐15 treatment affected follicular quantification after transplantation. Through follicular quantification data on day 6 after mice autologous ovarian transplantation, animals treated with BGP‐15 had no significant differences related to the number of primordial follicles (A, *p* > 0.05). Significantly, the ovaries showed a higher number of transitional (B, *p* = 0.0134) and primary (C, *p* = 0.0148) follicles than those of animals that received only vehicle. On the other hand, the count of secondary follicles was lower in the BGP‐15 group (D, *p* = 0.0441), while antral follicles did not demonstrate statistical differences (E, *p* > 0.05). Statistical differences were also observed regarding the number of atretic follicles (F, *p* < 0.0001). On day 22, follicular quantification demonstrated no statistical differences regarding primordial (G, *p* > 0.05), but a greater number of transitional (H, *p* = 0.0206), primary (I, *p* = 0.0125), secondary (J, *p* = 0.0428) follicles, with the BGP‐15 group having a higher count compared to the Vehicle. No statistical differences were observed related to antral follicles (K). The number of atretic follicles (L) was lower in the BGP‐15 treated group (*p* = 0.0381). Histological sections from day 22 showed a large number of healthy follicles across different classes in both the BGP‐15 and Vehicle groups (M–R). A higher number of transitional (yellow arrowheads), primary (green arrowheads), secondary (red arrows) follicles, and antral follicles (orange arrows) was observed in the BGP‐15 group. Bars = 100 μm (M and P). Bars = 20 μm (N, O, Q, and R).

On the sixth day after transplantation, BGP‐15‐treated animals showed no statistically significant differences in the number of primordial follicles (*p* > 0.05; Figure 5A). An increased number of transitional (*p* = 0.0134, Vehicle 1.4 ± 0.1; BGP‐15 2.3 ± 0.2; Figure 5B) and primary (*p* = 0.0148, Vehicle 1.5 ± 0.1; BGP‐15 2.2 ± 0.3; Figure 5C) follicles, contrasting to a lower number of secondary (*p* = 0.0441, Vehicle 1.7 ± 0.3; BGP‐15 1.0 ± 0.0; Figure 5D) was observed in BGP‐15‐treated animals. The number of antral follicles was similar between treated and Vehicle (*p* > 0.05; Figure 5E). At the same time, the number of atretic follicles was reduced in BGP‐15‐treated ovaries (*p* < 0.0001, Vehicle 0.2 ± 0.1; BGP‐15 0.04 ± 0.02; Figure 5F).

On day 22, treatment with BGP‐15 did not alter the number of primordial follicles (Figure 5G; *p* > 0.05). Statistical differences were observed between the groups, with emphasis on the the BGP‐15 treated animals, presenting a greater number of transitional (*p* = 0.0206, Vehicle 1.5 ± 0.15; BGP‐15 2.7 ± 0.4; Figure 5H), primary (*p* = 0.01, Vehicle 1.5 ± 0.1; BGP‐15 2.1 ± 0.2; Figure 5I) and secondary follicles (*p* = 0.04, Vehicle 1.4 ± 0.2; BGP‐15 2.7 ± 0.5; Figure 5J). The number of antral follicles was not altered by treatment with BGP‐15 (*p* > 0.05 ‐ Figure 5K). Atretic follicles were reduced on the BGP‐15 treated animals (*p* = 0.0381, Vehicle 3.4 ± 0.4; BGP‐15 2.6 ± 0.19; Figure 5L). Histological sections demonstrated that the ovaries treated with BGP‐15 contained healthy follicles in different classes, as well as a reduced number of atretic follicles (Figure 5M–R).

### 2.5 Collagen Deposition in Transplanted Ovaries was Reduced by the BGP‐15 Treatment

**Figure 6:**
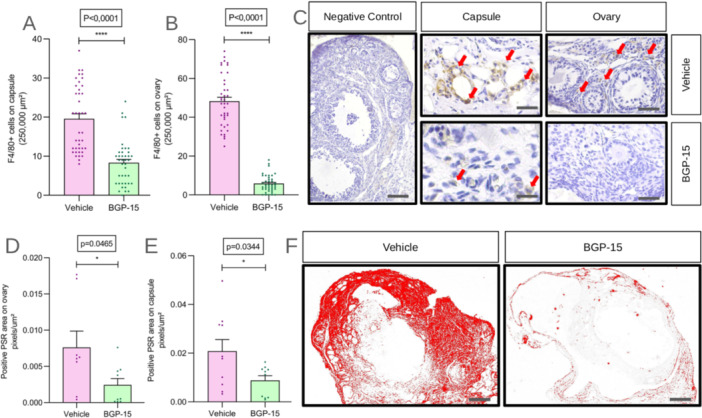
Treatment with BGP‐15 affected the number of macrophages in transplanted ovaries. Animals treated with the drug showed a lower number of F4/80+ macrophages per 250,000 μm^2^ in the capsule (A, *p* < 0.0001) and in the ovary (B, *p* < 0.0001). Histological sections illustrate these findings (C). Negative control bars = 200 μm, Capsule bars = 20 μm, Ovary bars = 100 μm. In vivo administration of BGP‐15 altered collagen deposition in transplanted ovaries. BGP‐15‐treated animals showed a lower positive PSR area (pixels/um^2^) both in the capsule (D, *p* = 0.0344) and ovary (E, *p* = 0.0465). The histology also clearly demonstrates that the BGP‐15‐treated animals showed a lower area stained with Picrosirius compared to the Vehicle samples (F). Bars = 200 μm.

BGP‐15‐treated animals showed a reduced number of macrophages per 250,000 μm^2^, both in the capsule (*p* < 0.0001, Vehicle 19.50 ± 1.3; BGP‐15 8.3 ± 0.9; Figure 6A) and in the ovary (*p* < 0.0001, Vehicle 48.2 ± 2.1; BGP‐15 5.7 ± 0.7; Figure 6B). Figure 6C illustrates F4/80 immunostaining. BGP‐15‐treated animals showed a reduced PSR positive area (pixels/um^2^) compared to the Vehicle, considering capsule + ovary (*p* = 0.0344, Vehicle 0.02 ± 0.05; BGP‐15 0.01 ± 0.001; Figure 6D) and only in the ovary (*p* = 0.0465, Vehicle 0.007 ± 0.002; BGP‐15 0.002 ± 0.001; Figure 6E). Figure 6F qualitatively demonstrates the reduction of positive collagen area in the ovaries of treated animals compared to the vehicle.

### 2.6 Treatment With BGP‐15 Increased the Number of Blood Vessels in Transplanted Ovaries

**Figure 7:**
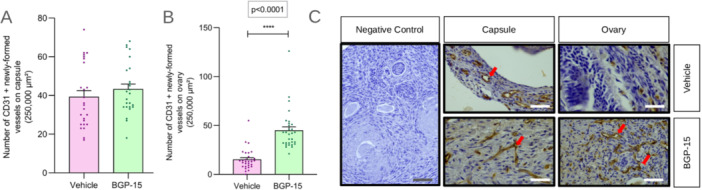
BGP‐15 administration affected the number of blood vessels after ovarian transplantation. No statistical differences were observed in the number of capsule CD31‐positive cells (A). BGP‐15 samples had a higher number of CD31‐positive cells per 250,000 μm^2^ in the ovary than Vehicle samples (B, *p* < 0.0001). Histological sections show a higher number of CD31‐positive cells per 250,000 μm^2^ in the ovary of the BGP‐15 samples (C). Negative control bars = 100 μm, Vehicle and BGP‐15 bars = 20 μm.

Immunohistochemistry for CD31 did not show statistically significant differences in the capsule (*p* > 0.05; Figure 7A). Still, it showed a difference in the number of blood vessels per 250,000 μm^2^ between BGP‐15‐treated and Vehicle‐treated animals in the ovary (Figure 7B). Transplanted ovaries from BGP‐15‐treated animals showed a higher number of blood vessels compared to the vehicle (*p* < 0.0001, Vehicle 39.2 ± 3.3; BGP‐15 43.2 ± 2.7). Figure 7C shows CD31 immunostaining, indicating a higher number of CD31+ blood vessels in the ovaries of BGP‐15‐treated animals than in Vehicle‐treated animals.

## 3 Discussion

This study demonstrated that the initial days post‐transplant are crucial for the occurrence of growing follicle depletion (Nascimento et al. 2023; Roness and Meirow 2019). This follicle loss occurs in parallel with increased collagen deposition, reinforcing the tight interconnection between structural remodeling and follicular dynamics during graft adaptation. As previously demonstrated by our research group, the early post‐transplant period is critical for follicular survival, with the most pronounced loss of growing follicles occurring between days 3 and 5 after transplantation (Nascimento et al. 2023). In agreement with these findings, the present data revealed a marked reduction in the follicular population between days 1 and 5 post‐transplantation, as evidenced by follicular quantification. Notably, secondary follicles were no longer detected from day 4 onward, reinforcing the severity of follicular depletion during this critical interval. In the field of fertility preservation, new drugs must be evaluated to modulate follicle survival and growth, thereby improving the outcomes of ovarian transplantation. In this regard, this paper demonstrates, for the first time in the literature, that BGP‐15 treatment reduces collagen deposition and stimulates follicle growth in transplanted ovaries. Because BGP‐15 is a new, unapproved FDA drug, our laboratory‐based study clearly demonstrates that additional clinical studies are needed to evaluate its efficacy and safety.

Postnatal Day 3 (PD3) mouse ovaries were used because this stage marks a critical period for germ cell cyst breakdown, primordial follicle formation, and early follicular activation. In mice, these events occur predominantly between birth (PD0) and PD3–PD4, making PD3 a widely used model for studies of early follicular recruitment. Previous studies investigating the PI3K/AKT pathway have successfully employed PD2–PD4 ovaries to evaluate primordial follicle activation and early follicular growth (Reddy et al. 2008; Adhikari et al. 2009; Hsueh et al. 2015). This methodology was also previously standardized by our group (Oliveira et al. 2026). Therefore, PD3 ovaries were considered an appropriate experimental model for evaluating early follicular activation and responses to pharmacological interventions.

The dose‐response curve showed that the lowest dose (0.001 μM) yielded the most promising results for primordial follicle activation. To our knowledge, this is the first study to report the efficacy of BGP‐15 in inducing follicle activation in vitro, opening new perspectives for the pharmacological manipulation of this process. Using follicular quantification, we observed that BGP‐15, at low concentrations, induced activation of primordial follicles in neonatal ovaries. However, gene expression analyses demonstrated upregulation of Pten, which would be expected to inhibit the PI3K/AKT pathway and prevent follicular activation. This apparent paradox suggests that BGP‐15 promotes follicular activation through alternative mechanisms.

Sirtuins have recently emerged as key regulators of female mammalian reproduction due to their role in folliculogenesis, oocyte maturation, and early embryonic development (Tatone et al. 2018; Yang et al. 2018). In this context, BGP‐15‐treated ovaries showed upregulation of Sirt1 and Sirt3, which may explain the increased number of activated follicles observed. Sirt1 has been described as promoting primordial follicle activation, including through modulation of downstream pathways such as AKT and mTOR (Zhang et al. 2017; Zhang et al. 2019), while Sirt3 plays a central role in regulating mitochondrial function and reactive oxygen species (ROS) levels within oocytes (Fu et al. 2014). Together with the observed modulation of NAMPT, these findings support the hypothesis that BGP‐15 induces follicular activation through a metabolic axis involving SIRT1/SIRT3‐dependent regulation of cellular bioenergetics and redox balance; however, further studies are needed to elucidate how mitochondrial activation triggers this process.

The ovaries analyzed in Figure 3 were not treated with BGP‐15 and were used to characterize follicular atresia during the early post‐transplantation period. A marked reduction in growing follicles, including secondary follicles, was observed after transplantation, consistent with ischemia‐induced follicular loss caused by the absence of immediate vascularization, which primarily affects metabolically active follicles (Tavana et al. 2017; Wu et al. 2024). These findings demonstrate the depletion of growing follicles in this model and support the results presented in Figure 5, which show that BGP‐15 treatment was associated with improved survival of growing follicles. Despite the increased follicular activation observed in vitro and after BGP‐15 treatment, 6th‐day follicle quantification showed that treated animals had fewer follicles. This apparent discrepancy likely reflects the dominant effect of ischemia during the early post‐transplantation period, which may override the beneficial effects of follicular activation. At this stage, neovascularization is still poorly established, compromising follicular survival despite treatment (Oktay et al. 2021). Ischemia is the main factor responsible for massive follicular loss in ovarian transplants, as the absence of blood supply and hormonal support leads follicles to atresia. Accordingly, 6 days is insufficient for complete revascularization, resulting in significant follicular depletion (Van Eyck et al. 2009; Mahmoodi et al. 2014; Nascimento et al. 2022).

Primordial follicles, due to their quiescent state and lower metabolic demand, are more resistant to ischemic stress and may contribute to the re‐establishment of the follicular pool once revascularization occurs. Given that folliculogenesis takes approximately 21 days following activation (Tavana et al. 2017; Wu et al. 2024), later time points better reflect graft recovery. In this context, although BGP‐15‐treated animals tended to show more morphologically normal follicles and fewer atretic follicles, these findings should be interpreted with caution. Rather than indicating a direct pro‐angiogenic effect, the data suggest an association with improved vascular network and follicular survival.

At later time points, when revascularization is more established, the effects of BGP‐15 become more evident. On day 22, treated animals showed reduced follicular atresia, associated with an increased number of blood vessels (Oktay et al. 2021; Bedoschi et al. 2013; Rajabzadeh et al. 2020). This improvement likely enhances follicular survival by reducing hypoxia‐related damage. BGP‐15 has been described as a mitochondrial activator (Gonzalez et al. 2024), and mitochondrial function is closely linked to ovarian aging and maintenance of the follicular reserve (May‐Panloup et al. 2016). In this context, improved cellular metabolic resilience may contribute to both follicular survival and the establishment of vascular networks within the graft. These findings should be interpreted in light of the well‐established vulnerability of ovarian grafts to ischemic injury. Substantial follicular loss and increased atresia are consistent with post‐transplant follicular depletion (“burnout”), with 50%–90% of follicles lost during the early post‐grafting period due to ischemia and premature activation (Cacciottola et al. 2021).

The immediate post‐transplantation phase is characterized by a hypoxic environment that persists until neovascularization is established, during which growing follicles are particularly susceptible to atresia. In contrast, primordial follicles exhibit greater resistance due to their reduced metabolic activity (Roness and Meirow 2019). Transplantation itself may induce premature activation of primordial follicles through pathways such as PI3K/AKT and mTOR, accelerating depletion of the follicular reserve (Chu et al. 2025). In this context, the increased follicular activation observed in our ex vivo model, together with improved survival of growing follicles after transplantation, suggests that the treatment may facilitate replenishment of the growing follicle pool following revascularization. This reinforces the concept that modulation of follicle activation dynamics is a key strategy to mitigate ischemia‐induced follicular loss.

Finally, ovarian aging is associated with fibrosis driven by macrophage infiltration and excessive collagen deposition, negatively affecting follicle number and quality (Umehara et al. 2022). In addition to its metabolic effects, BGP‐15 treatment reduced macrophage infiltration and collagen deposition while promoting follicular growth, corroborating previous findings in aged and obese models (Umehara et al. 2022). Notably, this study employed a lower dose and a different route of administration, highlighting the need for further in vivo studies to better characterize the pharmacological and pharmacokinetic properties of BGP‐15 in ovarian tissue. Overall, our data indicate that BGP‐15 exerts multifaceted effects: in vitro, it stimulates follicle activation and modulates metabolic pathways; in vivo, it promotes follicle growth, reduces atresia, improves tissue structure, and increases the number of blood vessels in transplanted ovaries. Collectively, these findings support a model in which BGP‐15 improves ovarian graft resilience by integrating metabolic regulation, modulating follicular activation dynamics, and enhancing the local microenvironment, thereby promoting follicular survival after transplantation.

## Author Contributions

**Luiza Aparecida Ansaloni Chagas Pereira:** conceptualization, investigation, writing – original draft, methodology, validation, writing – review and editing, software, formal analysis, project administration, data curation, visualization. **Larissa Aline Freitas:** validation, formal analysis. **Camila Stefane Ferreira:** writing – review and editing; methodology, validation, formal analysis, data curation. **Karine Stephany Serpa Amaral Dias:** methodology. **Paulo Henrique Almeida Campos‐Junior:** conceptualization, investigation, funding acquisition, writing – original draft, methodology, validation, visualization, writing – review and editing, software, formal analysis, project administration, data curation, supervision, resources.

## Ethics Statement

The Ethics Committee for the Use of Animals of the Federal University of São João del‐Rei (protocol n° 6986150322) approved all procedures.

## Conflicts of Interest

The authors declare no conflicts of interest.

## Data Availability

The data that support the findings of this study are available on request from the corresponding author. The data are not publicly available due to privacy or ethical restrictions.
